# Purpose in Life in Older Adults: A Systematic Review on Conceptualization, Measures, and Determinants

**DOI:** 10.3390/ijerph19105860

**Published:** 2022-05-11

**Authors:** PV AshaRani, Damien Lai, JingXuan Koh, Mythily Subramaniam

**Affiliations:** 1 Research Division, Institute of Mental Health, 10 Buangkok View, Singapore 539747, Singapore; mythily@imh.com.sg; 2 Geriatric Psychiatry, Institute of Mental Health, 10 Buangkok View, Singapore 539747, Singapore; damien_pl_lai@imh.com.sg; 3 Lee Kong Chian School of Medicine, Nanyang Technological University, Clinical Sciences Building, Singapore 308207, Singapore; kohj0056@e.ntu.edu.sg; 4 Saw Swee Hock School of Public Health, National University of Singapore, Singapore 117549, Singapore

**Keywords:** purpose in life, older adults, conceptualization, measures, determinants

## Abstract

Purpose in life (PIL) is a psychological construct that reflects one’s life goals and the desire or determination to pursue them. Having a purpose provides an intrinsic motivation to adopt healthy behaviors as we age, which will help us to achieve positive health outcomes. Thus, promoting PIL is the cornerstone for successful aging and better health outcomes. This systematic review aims to identify how PIL is conceptualized, measured in the existing literature and what are the determinants of PIL in older adults (≥65 years). Electronic searches were conducted in five databases (Medline, PsychInfo, Embase, CINAHL and Web of Science). A total of 44 studies were included in the review. PIL was conceptualized in six different ways: health and well-being, meaningful goals and purpose, inner strength, social relationships, mattering to others, and spirituality and religiousness. There were six main questionnaires and semi structured interviews used to capture PIL. Female gender, higher education and income, being married, ethnicity, health and well-being, inner strength, social integration and spirituality were associated with PIL. Majority of the included studies had low to moderate Risk of Bias (RoB) assuring confidence in the results. The conceptual frameworks of PIL identified in the review underscore the complexity of the construct. Several sociodemographic and other determinants of PIL were identified.

## 1. Introduction

Purpose in life (PIL) is a psychological construct that contributes towards positive well-being [1]. Viktor Frankl [2,3], the pioneer psychiatrist who proposed the concept of PIL based on his personal experiences in the concentration camps during Holocaust, proclaimed that the pursuit of meaning in life (MIL) is central in one’s life. He conceptualized PIL as a pathway to achieve MIL and noted that the realization of having a PIL was protective against suicide and helped one to face the challenges of daily life. Frankl also emphasized in his book *Man’s Search for Meaning* that although the MIL changes in one’s life, from time to time, the need for a meaning still remains [3] thus making it a crucial construct in health promotion. PIL and MIL had no distinctive definition until the 1990s. Baumeister [4] further noted that the search for meaning involves four basic needs: need for a sense of purpose in life, self-worth, self-efficacy and value. Steger [5,6] further clarified these constructs where he suggested that MIL exists as two concepts: A cognitive component where the person understands who he is and how he fits the world around him and a motivational component that provides the individual with a purpose/goal in life. Reker [7] revised this and included an affective component that reflects that meaning is incomplete without individual satisfaction. Costin and Vignoles [8] in their longitudinal study, assessed the temporal relationship between purpose, coherence, mattering and MIL judgments and evidenced that while mattering is a precursor for MIL judgement, others were not. George and Park [9] studied the distinction between PIL and MIL constructs in a longitudinal sample of cancer survivors and noted a clear distinction between the constructs in terms of their predictors and correlates, further confirming the above argument. Damon et al. [10] reviewed various definitions of PIL and MIL and proposed that PIL has a more specific role that is not captured in the broader definition of MIL. The authors defined PIL to include the three characteristics (a) having a stable far fetching goal; (b) a desire to achieve something meaningful beyond self; and (c) making progress to accomplish the goal. Thus, PIL is considered as one of the distinct components of MIL. Meaning sometimes, not always, drives the development of purpose, however when PIL is established, it helps in the development of MIL [11]. Thus, PIL and MIL are distinct constructs with temporal bidirectional relationship.

PIL gathered much interest among health practitioners in recent years due to potential in promoting health outcomes. Smith and Zautra [12] showed that PIL is related to less depression, anxiety and other negative effects thus promoting recovery of the patients. A systematic review of 10 studies showed that a high score on PIL was associated with 17% reduced risk of all-cause mortality and cardiovascular events [13]. Likewise, a greater PIL was predictive of a lower allostatic load at 10 year follow up [14]. Similarly, studies have shown a relationship between a lack of PIL to depression, substance use and self-derogation where women tend to use substances and males expressed higher suicide ideations [15]. Another study conducted in a psychiatric sample showed that PIL mediated the relationship between, depression, life satisfaction and suicide ideation [16]. PIL showed higher protective effect against suicide ideations when depressive scores were higher [16]. Aghababaei and Blachnio [17] reported that PIL predicted happiness and life satisfaction in a Polish sample of university students. PIL is positively associated with well-being and positive emotions [18]. In agreement with Frankl’s theories [2,3], other studies have also confirmed that the existential vacuum created by the lack of PIL is the root for psychological disorders.

While development of PIL is a lifelong process, studies evidenced that PIL tends to drop with old age [19]. Employment provides an avenue for social integration and identity while retirement changes that structure leading to an existential crisis [20]. This observation was confirmed by a meta-analysis that showed a negative correlation between retirement and PIL [19]. Yemiscigil and colleagues [20] reanalyzed the data from national health and retirement survey and confirmed the above relationship. Nonetheless a quasi-experimental analysis by the authors that took into account limitations of correlation analysis showed a positive causal relationship between retirement and PIL, especially among those with low socioeconomic status, in their early retirement years. Retirement, chronic illnesses and widowhood also were associated with a decline in PIL [21]. Evidence suggests that having a PIL protects against cognitive decline [22], Alzheimer’s disease [23], disability [24], cardiovascular complications [25] and death [26] in older adults. Older adults who have higher PIL engage well with life leading to higher life satisfaction, and greater well-being in life [27]. Additionally, older adults who have a sense of purpose value their life and adopt a healthy lifestyle paying attention to their diet, exercise and physical health [28]. A systematic review and meta-analysis of 70 studies looked at PIL in middle and older age and reported an age specific decline in PIL [19]. Thus, helping the older adults to develop or maintain a PIL is imperative given the rapidly aging population across the globe. To achieve this, it is of paramount importance to understand how PIL is conceptualized (defined and understood). The constructs that are identified can potentially lead to interventions that can be implemented in the wider population. It will also help in choosing appropriate tools for measuring the constructs comprising PIL in this population while designing interventional studies. Currently, many studies use the concepts of PIL and MIL interchangeably with no clear distinction, which makes the data heterogeneous and redundant. This information will be crucial to intervene and promote/maintain PIL in community-dwelling older adults who often undergo purpose derailment during retirement or widowhood resulting in adverse psychological outcomes. Systematic reviews have been conducted to look at MIL, its effect on physical health [29], and instruments used to measure the construct [30]. The last systematic review on PIL published in 2002 looked at age specific decline in PIL, association of socioeconomic status, gender, and psychological well-being with PIL. Irving et al. [31] conducted a systematic search and review to understand the physical and psychosocial impacts of PIL and its correlates in people above 55 years. These two reviews had different focus and population. This systematic review is timely given the last systematic search and review was published in 2017 and the field has grown considerably in the past few years. Moreover, the previous reviews did not differentiate between articles that discussed PIL from MIL and thus this review provide a new direction. No systematic reviews have been conducted till date to assess how PIL is conceptualized, measured and the factors associated with PIL in those aged 65 years or above. The current review included all study designs (quantitative and qualitative data) and summarized the evidence on how PIL is conceptualized, measures used to capture PIL in the older adults (≥65 years), and the factors associated with PIL in older adults

## 2. Materials and Methods

This review followed Meta-Analysis of Observational Studies in Epidemiology (MOOSE) guidelines [32] of reporting. The review included both qualitative and quantitative studies which were integrated using a convergent integrated approach. The quantitative data were transformed to qualitative description and were analyzed and synthesized together with qualitative data [33]. This was done by collecting the quantitative and qualitative data to the data collection forms and integrating the results by grouping the quantitative information to themes using thematic analysis. The study protocol for this review is not registered, however, it is available upon request.

### 2.1. Eligibility

Studies were included if they investigated the PIL in community-dwelling older adults. Older adults were defined as those who were ≥65 years [34]. Clinical cohorts and those who were living in nursing homes were excluded unless they were used as a comparison group against community-dwelling adults. The studies were grouped according to three outcomes for synthesis. All studies involving primary data were included. Studies were excluded if they reported unrelated outcomes, conducted in animal models, involved clinical cohorts (e.g., terminally ill patients, as they might have a different perception of PIL than community-dwelling individuals) or were published before 1995 or in a language other than English. Additionally, case series, case reports, conference abstracts and reviews were excluded. We contacted two authors for clarification regarding the methodology, however, we did not get any replies. These studies (n = 4) were excluded as eligibility could not be determined. Grey literature and thesis were included in the review which were searched using Google Scholar and OpenGrey database. To make the review more focused, articles which used the term MIL without an explicit reference to PIL (not reporting the data on purpose as a distinct construct) were excluded.

### 2.2. Information Source and Study Selection

Five databases (Medline (Ovid), PsychInfo (Ovid), Embase, CINAHL, and Web of Science) were searched electronically for articles published during the period of 1995 until the present. A detailed list of keywords employed for each database has been included in the Supplementary File S1, Section S1. Additional sources included bibliographic searches and grey literature that captured thesis and reports, which do not appear in regular searches. The review (title, abstract, and full text screening) was independently conducted by any two reviewers (A.R., M.S., D.L. or K.J.X.) and disagreements were resolved by further discussion or by the third reviewer. Authors were contacted for Supplementary Information to decide the inclusion or exclusion of articles. The search was updated on 1 November 2021.

### 2.3. Risk of Bias (Rob) and Quality Assessments

Methodological bias was assessed using the Joanna Briggs Institute (JBI) critical appraisal checklist by the three reviewers. For the mixed methods study, the Mixed Methods Appraisal Tool (MMAT) was employed. Studies were classified into three risk groups: low, medium and high RoB following the cut offs recommended by Goplen et al. [35] Briefly, studies were classified to have low RoB if they scored the answer ‘yes’ for 70% of answers, moderate RoB if 50–69% and high RoB if they scored below 49%. GRADE (Grading of Recommendations Assessment, Development and Evaluation) approach was not employed in the study to assess the quality of evidence as majority of the studies included were either cross sectional or cohort studies which start with a low quality of evidence due to the innate limitations in the study design [36]. GRADE-CERQual was used to assess the confidence in qualitative synthesis. A detailed table with the RoB of included studies is included in Supplementary File S1, Table S1.

### 2.4. Data Extraction and Synthesis

A pilot data extraction form was developed based on our previous systematic review [37] and tested using two studies by all three reviewers. All three reviewers extracted the data, and one reviewer cross checked the extracted data and highlighted any discrepancies for discussion and resolution. The extracted data included, first author and year of publication, title of the study, aim of the study, population characteristic (any specific age group, gender), study settings, sample size, study design, country where the data was collected, methods, outcome measures used and main results. 

The main outcomes synthesized in this review are conceptualization, which provide a systematic analysis of the use of the term in literature to identify the common understanding of the concept, the measures used to capture the concept, which includes questionnaires that captured PIL (type of instrument used, number of items, psychometric properties) and their coverage to the concepts identified and finally the determinants of PIL in elderly. Studies were grouped according to the outcomes and data were synthesized narratively. 

The conceptual framework for PIL was carried through systematic search and content analysis as reported by Rajabzadeh et al. [38] to get a clear understanding of PIL. The included 43 articles were searched for texts defining PIL in all parts of the manuscript. One article (thesis) was excluded [39] from this analysis as there was a subsequent article [40] by the same authors that was included in the analysis, which reflected the same concepts. Hence, to avoid duplication the peer reviewed article was included in the conceptualization analysis. Content analysis was performed and the texts were coded by two reviewers. The codes were further re-grouped into themes/higher order categories, which were revised to give the final framework. The initial framework was constructed using 20 articles and validated by vote counting to ensure the stability of the framework [38]. 

The measures used to capture PIL were grouped according to the type/name of measure, number of items, and psychometric properties. The determinants were captured by analyzing the respective text and capturing the determinants in textual form.

A narrative synthesis was employed instead of meta-analysis, to summarize the data as the analysis of the primary outcomes (conceptualization and measures used) were qualitative in nature. Secondary analysis of data from larger studies was highlighted and not combined. Studies that included cross sectional data from large longitudinal design (e.g., Umea 85+ study (6 articles, mixed methods longitudinal study)), Health and Retirement Study (2 articles, data from different time points), National Health and Aging Trends Study (2 studies, one used data from 2011 wave the other is cumulative data) were treated as separate studies if they employed distinctive cross-sectional datasets.

## 3. Results

### 3.1. Study Characteristics

**Figure 1:**
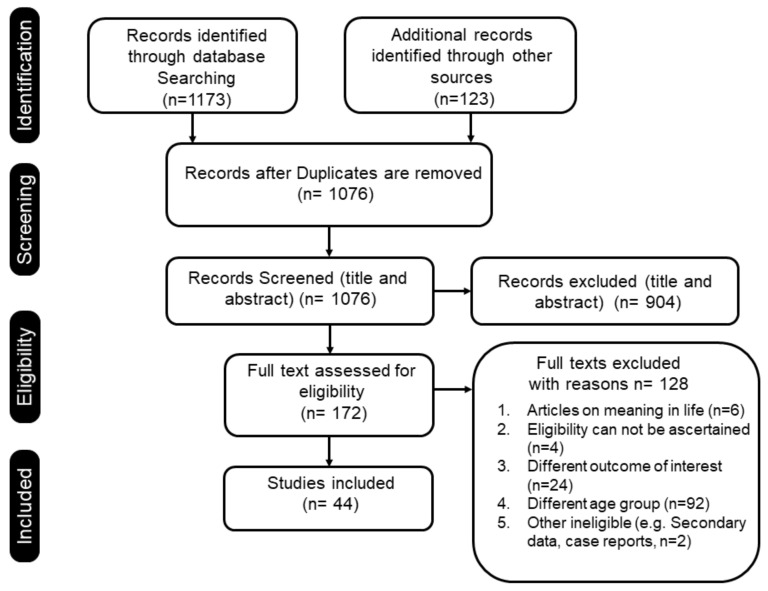
Database search and number of included studies.

A total of 1173 records were retrieved from the searches of the five databases and 123 from other sources. After removing the duplicates, 1076 articles were selected for title and abstract screening. Of these 44 articles were eligible and were included for data extraction. The details of the process are indicated in Figure 1. Of the total 44 articles included in the current systematic review, 19 were from the US, seven from Sweden, four from Australia, two from France, three from Japan and nine from other countries. Among the included studies 28 were cross sectional, 12 were observational cohorts 3 were qualitative studies and 1 was a mixed methods study (Table 1). 

### 3.2. Conceptualization of PIL

**Figure 2:**
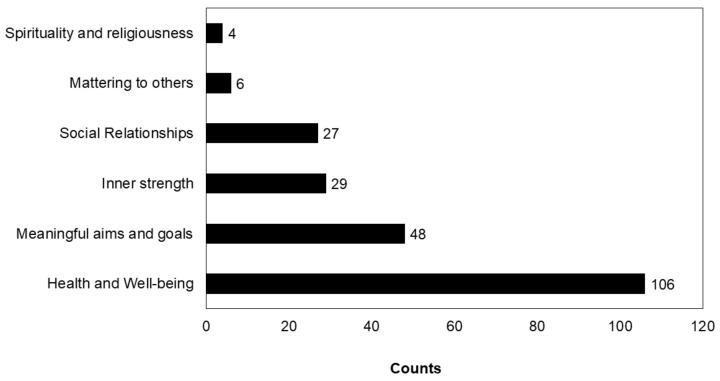
Conceptualization of PIL. The bars represent the frequency of votes.

Six main concepts were derived through content analysis: spirituality and religiousness, health and wellbeing, meaningful aims and goals, social integration, mattering to others and inner strength. Of the 41 articles included (one was excluded and the 2 articles did not offer any information), there were 106 counts for health and well-being (Figure 2), followed by meaningful aims and goals (n = 48) inner strength (n = 29), social relations (n = 27) mattering to others (n = 6) and spirituality and religiousness (n = 4). Studies adopted more than one conceptualization of PIL (Table 2). The details of the domains, subdomains and the coverage are reported in a later section.

Majority of the votes fall under the health and well-being domain which was broadly classified into overall well-being (n = 6), mental (n = 40) and physical health (n = 60). Fourteen studies described PIL as psychological well-being/better mental health while 19 studies portrayed PIL as having a good health status (includes overall health, physical functioning and lower disabilities). Thirteen studies reflected the subtheme of ‘adapt to the body’, which included positive attitudes towards aging, finding peace with declining health and acceptance of death. Nine studies narrated the concept of longevity/reduced mortality while 8 studies included cognition. PIL was also reflected as eudaimonic well-being (n = 8) and absence of depression (n = 7).

Out of the 48 votes reflecting meaningful aims and goals, 16 indicated values driven goals/goal directedness, 8 showed direction in life, 4 each included role identity and looking forward to the future. Other minor sub themes included engagement with challenges, growth, personal growth, existential vacuum and autonomy. The major sub themes contributing to the concept of inner strength were resilience (n = 6), self-esteem (n = 5), perception of control (PC, n = 5) and sense of coherence (SC, n = 3). The minor subthemes were self-acceptance, self-improvement, personal strength, optimism, competence, coping strategy and self-realization. Social networks (n = 8) and positive relationships gathered the majority of the votes under social relationships. The sub themes included social integration, absence of loneliness, self-respect, happy marriage or having family, support and care from others and social participation. Among the six votes captured for ‘mattering to others’, four included the concept of living an honorable life which included helping others and being good to others, and two included making a difference in someone’s life. Spirituality and religion gathered a total of four votes.

### 3.3. Measures of PIL

Multiple measures including specific validated scales, modified versions, subscales of larger scales, and single items, were used to capture PIL. In total six questionnaires were used and 3 studies used single item measures to capture PIL. The most common measures were PIL subscales of Ryff’s Psychological Well-Being Scale (Ryff’s PWB scale; n = 20), followed by PIL test by Crumbaugh and Maholick [81] (n = 10, PIL Test-C), NIH Tuberculosis Meaning and Purpose Scale Age 18+ (n = 2), Life Engagement Test (LET, n = 2), subscale from meaning in life scale (n = 1) and K-1 scale (n = 1) and other modified versions (n = 6, Table 1). A detailed list of various measures employed in the included studies, main results and psychometric properties are included in Supplementary File S1, Table S2.

#### 3.3.1. Ryff’s Psychological Well-Being Scale (Ryff’s PWB Scale)

Ryff’s PWB scale measures well-being under six domains: autonomy, environmental mastery, personal growth, positive relation, PIL and self-acceptance [82]. Over the years several modifications of the scale had been studied which varied from 14, 10, 9, 8, 7, 6, 3 and single item versions. However, the validity and reliability of these versions remained contentious [83]. Moreover, the validity of the PIL subscales when used independently and in new settings and populations remains debatable. Twenty studies included in the review used the PIL domain of this scale either as a subdomain of well-being or independently to measure PIL. Another three studies used modified versions of the scale (Supplementary File S1, Table S2). Kim et al. [26] created a modified version of the Ryff’s PWB scale by combining five items from psychological well-being and two additional items from personal growth (PG) and acceptance which the authors validated in the study population (α = 0.76). Sutin et al. [71] examined PIL of older adults using secondary data from two separate large-scale studies. The authors combined a seven-item PIL subscale from Ryff’s PWB scale (reliability 0.74) and a single item measure, the psychometric properties of which were not clearly stated. Bundick et al. [77] employed a new measure comprising 10 items combined with 9 items from Ryff’s PWB scale. All these different versions employed different scoring and interpretation criteria.

#### 3.3.2. Purpose in Life Test (PIL Test)

The PIL Test was developed by Crumbaugh and Maholick [81,84] supporting Frankl’s concepts, and measures PIL and MIL. The scale has been widely used in different settings and populations [85]. It includes three parts: Part A, a 20-item questionnaire scored on 7-point Likert scale, a part B including 13 sentences and an open-ended part C where the participant indicates the progress, they have made in achieving their life goals. Nonetheless, only Part A is used in the final analysis where a higher score indicates higher PIL. Scores above 112 indicate definite PIL, 92–112 shows indecisive stage and below 92 indicates no clear purpose. Ten studies were included this scale. The scale had high reliability, split half reliability (ranging from 0.77–0.85), and test retest reliability (0.68–0.83). Alpha coefficients ranged from 0.86 to 0.9 [85,86]. The scale also showed adequate face, construct, construct-convergent, construct-discriminant and criterion-concurrent validity [85]. Thus, the PIL test meets the criteria for acceptable level of psychometric properties. Moreover, the scale employed similar scoring criteria in all included studies.

#### 3.3.3. NIH Tuberculosis Meaning and Purpose Scale Age 18+

The scale included 18-items (three domains) and has been validated in adults [87] but not in older adults. Two articles included in the review employed this tool. One study [66] used an adapted 7-item version of the subscale to measure PIL with no data on validity and reliability while another study reported a Cronbach α of 0.93 [72]

#### 3.3.4. Life Engagement Test (LET)

LET measures PIL as the extent to which the person values the life goal and engages in tasks to achieve the goals. This is a 6-item scale (five response options) with moderate stability as indicated by test-retest reliability (correlations ranged from 0.61 to 0.76) Cronbach’s α ranges between 0.72 and 0.87 [27] with correlation of 0.73 with Ryff’s PWB scale (9-item). A higher score in the scale indicates higher PIL.

#### 3.3.5. K-1 Scale

K-1 scale includes 16 items under four domains (self-realization and will, sense of life fulfillment, sense of existence and will to live, scored on a 3-point scale. The scale had adequate validity [63], but not in older adults. 

In total six studies that employed Ryff’s PWB scale did not provide any information on the psychometric properties of the employed scale whereas all ten studies from PIL test [84] had information on either reliability, validity or both. The LET, K-1 and NIH tool kits had their psychometric properties reported.

Four studies included in the systematic review employed semi structured interviews to capture the barriers, contributors and experiences of PIL in elderly [50,57,70,74].

Out of the six scales used to measure PIL, none captured the concepts of spirituality, mattering to others and social relationships. Inner strength and meaningful aims were covered by the K-1 scale, while the later was captured by meaning and PIL subdomain of NIH toolbox, PIL test and LET. Health and well-being were captured by Ryff’s PWB scale. While the majority of the scales relied on the concept of meaningful aims and goals to capture PIL, other concepts that are relevant in older adults received no/minimal attention.

### 3.4. Determinants of PIL in Older Adults

#### 3.4.1. Sociodemographic Determinants

**Figure 3:**
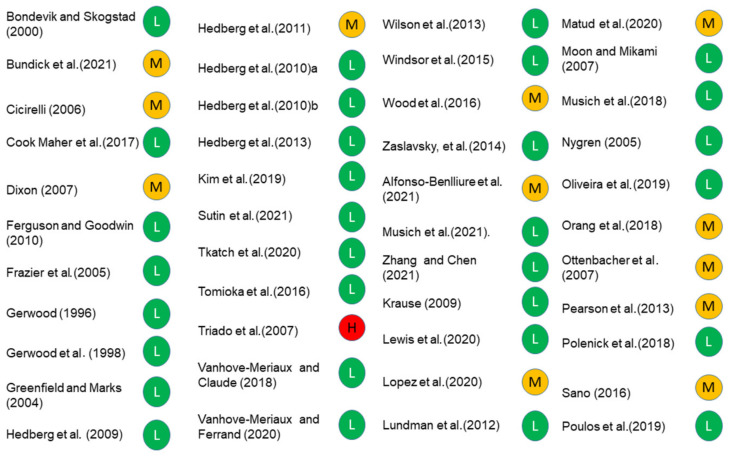
RoB of included studies.

Of the 34 studies that reported determinants of PIL, six reported sociodemographic determinants. Age, gender, education, income, ethnicity and marital status were associated with PIL (Table 3). Two studies [77,80] reported older age groups to have better PIL while Triado et al. [47] reported a disagreement where younger age was associated with PIL. Three studies showed an association of female gender with PIL [56,77,80]. Four studies showed higher education to be associated with PIL [47,59,62,80]. Two studies each reported association of marital status (being married) [62,80], income [47,62]) and ethnicity [77,80] with PIL. While Zhang and Chen [80] reported an association of PIL with ethnicity (being white/Caucasian), Bundick et al. [77] showed an association with being white. All, except Triado et al. [47], scored low-moderate risk of bias (Figure 3). Overall, the evidence suggests female gender, marital status (married) higher education, and income are determinants of PIL.

#### 3.4.2. Other Determinants

Twenty-one studies examined the association of PIL with various aspects of health which included mental and physical health. Depression was the mental health condition that was most associated with PIL [39,46,55,60]. Cognitive functioning and memory shared an association with PIL [22,58,60,71]. A single study reported association of PIL with Smoking and alcohol consumption [62] and perceived mental health [44]. All, except Dixon [46], Woods et al. [62], and Hedberg et al. [55], scored low RoB while the latter had moderate RoB. PG, self-acceptance, autonomy, PIL, environmental mastery and positive relations are components of psychological well-being [88]. Three studies (moderate—high RoB [47,49,78] showed an association of these determinants with PIL. Overall, the strength of evidence (based on frequency and methodological quality) linking lower depression and cognitive functions to higher PIL was moderate while the rest of the determinants are weak and thus should be interpreted with caution.

Three studies with low RoB showed an association of PIL with lower disabilities [26,51,60] while four studies (low RoB) showed association of higher PIL with reduced mortality [26,51,60,61]. Seven studies (five with low and two with moderate RoB) reported health status to be associated with PIL [26,45,51,60,66,75,80]. Two studies [46,48] showed an association of well-being with PIL and life satisfaction [69,78]. Life satisfaction is a concept that is linked to hedonic well-being [89]. In summary, there is moderate evidence linking health to PIL.

Resilience, SC and self-transcendence were reported to be positively associated with PIL in four studies with resilience appearing in all four studies [44,56,66,75]. Other determinants that were associated with PIL included optimism and PC [52]. There is substantial evidence to support the association of resilience to PIL with three of the studies showing low and one moderate RoB. 

A total of four studies (low RoB) showed an association of spirituality and religiousness [39,40,41,43]. However, the evidence is not strong enough to confirm this association due to the low number of studies and the included studies being secondary analysis of the data [39,40].

Other factors that were associated with PIL were social participation, support, loneliness, role expectations in the community and having family. Six studies showed an association of social well-being with PIL [41,42,53,63,66]. The evidence supporting the association is promising, however further research is needed for a better understanding of this association.

Some other factors associated to PIL include mattering to others (perception that they are valued and are important to others; Dixon [46], fear of death [45], attitude towards aging [54] instrumental activities of daily living [61], motivation and self-determination (includes achievement motives, competence and relatedness) [63,65] high satisfaction/low thwarting need [65], emotional and physical caregiving difficulties [68], psychological need frustrations and satisfaction [73] and intensity of physical activity [80]. However, the evidence is not strong enough to substantiate the observed association. 

### 3.5. Summary of Evidence

Among the 44 studies included, 31 had low, 12 had moderate and one had high RoB. The source of bias for most of the studies were the confounding factors, their analysis and loss to follow up. Furthermore, the questionnaire used in some of the studies had unclear validity and reliability. Additionally, six different scales and several modified versions of the existing scales were used to measure PIL each with different scoring and interpretation criteria which makes comparisons challenging. Many studies had limited information on the psychometric properties of the scale in the study population. This caveat in the design involving multiple versions of questionnaires with unknown psychometric properties limits the comparison of results across studies to reach a meaningful outcome.

This systematic review did not adopt GRADE criteria for the quality of evidence due to the nature of study design (observational) which would downgrade the quality automatically making the grading meaningless. The majority of the studies were cross sectional in nature and none were interventional. The quality of evidence for the qualitative data were evaluated using CERQual (Confidence in the Evidence from Reviews of Qualitative research) which showed moderate confidence in the results due to moderate concerns over methodological issues (insufficient information on the participant selection or context).

## 4. Discussion

The current systematic review aimed to synthesize the literature from the past two decades to understand how PIL is conceptualized, measured and the determinants of PIL in older adults. The understanding of determinants can lead to the development of community level interventions to promote PIL in older adults ensuring active aging and their wellbeing 

Despite the abundance of literature on PIL in the past two decades, the understanding of the term has not been explored till date. Many authors have used PIL and MIL interchangeably [90] while emerging reports showed a clear distinction [91]. Such differences in conceptualization of the construct can lead to differences in measurement and operationalization which are sources of heterogeneity between studies. The review identified six different ways of conceptualization of PIL: spirituality and religiousness, health and well-being, social relationships, meaningful aims and goals, mattering to others and inner strength. While the majority of the literature used the concept of health and well-being and meaningful aims and goals, which were definitions based on Frankl’s concepts [3], a substantial number of votes were noted for the remaining concepts. We examined the coverage of all the identified concepts in the existing measures used in the included studies and found that the majority of the questionnaires for PIL captured the above two concepts whilst none of the measures captured spirituality, inner strength, social relationships and mattering to others. A possible explanation for this could be the differences in the population of focus in the research surrounding PIL. Studies of PIL in adolescents showed that adolescents who reported PIL described social support from friends and family which they integrate to their living [92]. Another study among adolescents examined the relationship between psychological distress, religion, PIL and social relationships showed that the latter two constructs mediated an inverse relationship between religiosity and psychological distress where people with higher psychological distress tend to use religion to cope with the distress through social support and achievement of higher PIL [93]. The current review focused on conceptualization of PIL in older adults (≥65 years) while the measures were developed in adults and adolescents. Given that there are six different ways PIL is conceptualized, it provides a clear understanding of the construct that will inform and guide development of new measures for PIL. Future research should accommodate the new concepts and avoid over reliance on the founding concepts, to suit the population and changing environments.

The review identified six different instruments that can be used to measure PIL in older adults. While Ryff’s PWB index measures PIL as a subscale where multiple versions of the scales and specific subscales for PIL were adopted by the studies with little information on the reliability and validity in the specific population, PIL Test-C is a 20-item scale with adequate psychometric properties that had been used consistently across the reported studies. This gives confidence in the results from these studies employing PIL Test-C, allowing a meaningful comparison. Many studies included in the review have used different nomenclature of the scale (e.g., Ryff’s PWB scale was reported as PWB index). The nomenclature was standardized by identifying the scales based on the items included and grouping the scales based on the standard name. LET is another promising scale that has acceptable reliability and validity in the current population. While Ryff’s PWB scale and PIL Tests-C have adequate psychometric properties on their own, they have different lengths (3–38 items) and the longer ones might be a burden for the elderly population. Thus, the appropriateness and comfort of the respondents should be considered while choosing the instrument. Furthermore, the understanding of PIL could be different among different age groups, especially in elderly as evidenced by the qualitative studies [50,70,74]. Thus, care should be taken to ensure that the questionnaire employed is appropriate for the specific population.

We have also noted different concepts of PIL among the studies indicating the complexity of the construct. The selection of the instruments is influenced by the definitions/conceptualization of PIL by the authors. Ryff’s six factor model was rooted on eudaimonic well-being and considered PIL as one of the factors contributing to psychological well-being [94] whereas, Crumbaugh and Maholick [81,84] developed the PIL test to measure meaning and PIL based on Frankl’s concepts of existential vacuum which if unaddressed may lead to frustration and noogenic neurosis [95]. Nonetheless, the two scales have a high correlation suggesting meaning in life is a predictor of eudaimonic well-being [96]. On the other hand, LET defines PIL as the extent to which a person engages in activities that he values and K-1 scale is based on the concept of ‘ikigai’ which captures what the person values and brings him joy. Thus, it is clear that different authors used different concepts of PIL in their studies which are sources of heterogeneity in studies that limits the generalizability of the data. Additionally, the scales were used by different studies across the globe in different populations, regardless of the cultural differences and other sociodemographic characteristics of the population understudy. The cultural sensitivity of the scales is unknown which needs to be considered while selecting the questionnaire in specific populations. 

The current systematic review identified several determinants which included sociodemographic determinants such as female gender, higher income, education and marital status which is in agreement with previous review [31]. These factors are known sources of PIL as they are indicative of a successful life and give rise to a perception that their life has been meaningful [19]. This relationship can be direct or indirect which are mediated through various activities. Likewise, education provides knowledge regarding life goals and how to achieve them successfully. Higher income provides resources that can be used to pursue the goals and provide avenues for various life activities that the older adult can make use to achieve the PIL [19]. Additionally, the availability of financial resources helps the older adult to integrate to their social environment by providing help to those in need who approach them [97] which in turn will give them a feeling of mattering to others. Mattering to others is related to PIL and mental wellbeing [46]. The current systematic review has noted this concept as an important factor contributing to PIL. Contrary to our results, Pinquart [19] proposed in his review that female gender is associated with low PIL compared to males, as females tend to have low psychological well-being due to low socioeconomic status, higher chance of being widowed, having chronic conditions and inability to perform daily functions compared to their males. However, the author had also cautioned that this hypothesis can be nullified in older males, the majority of whom are in the workforce as they tend to experience a loss of PIL upon retirement [98]. The studies in the current review did not differentiate the educational and occupational details of the older adults and thus a comparison is not possible. 

Social integration includes social relationships, participation, and family relations which were identified to be associated with PIL in this review. Social integration imparts a sense of belonging, a feel of being wanted, respected and loved which will intrinsically motivate the individual to engage in the social relationships that adds purpose to their life. Studies have shown that older adults value their family relationships more than their friends thus forming the primary source of support [19]. These social relationships are suggested to be beneficial for the older adults to engage in various activities that will contribute to their PIL [19]. Such interactions also have long term effects on health. Social relationships also influence health behaviors. This includes spouses monitoring and influencing the health behaviors thus promoting their partner’s health [99]. Thus, this will lead to a sense of responsibility where the person changes his/her health habits which in turn will improve their physical health and reduce mortality [100]. Understanding these factors and their influence on PIL could be useful in addressing PIL decline in elderly population.

Inner strength is a human resource that helps one to recover, heal and thus promotes well-being and health. It helps to move ahead in life against all adversities to find the meaning in life [101]. It is captured as resilience, sense of coherence, sense of control and self-transcendence were found to be associated with PIL in this review. PIL is also identified as one of the inner resources for inner strength. A higher inner strength is associated with better social integration and health in older people [56] and thus should be the focus of preventive strategies to tackle loss of PIL in the elderly.

Finally, health and well-being has been associated with PIL in current and previous reviews [19]. This involved both mental and physical health and overall well-being. Conditions such as chronic stress can lead to immune system dysfunctions where PIL motivates people and helps them to stay put without giving up, thus having direct effects on physical well-being. PIL promotes healthy lifestyle practices [28]. Having a purpose also deter individuals from harmful behaviors such as substance use and protects against mental health decline [102]. PIL helps individuals to experience pleasure from achievements and promotes a sense of achievement [19]. This will further reduce chances of depression thus promoting mental well-being. Furthermore, social integration and inner strength which are determinants of PIL were associated with well-being [19]. Hence, focusing on these factors will not only promote PIL, but also helps in improving the physical and mental well-being of the older adults.

Sociodemographic factors and various factors related to health (depression, memory, disability, health, substance use, etc.) were associated with PIL. Individuals often seek their purpose in life as early as their childhood which will strengthen during their transition to adulthood. During adulthood and midlife, people embrace the concept strongly and seek the purpose according to the various roles they play in their life and community [103]. However, during midlife, major changes happen in one’s life which include retirement, change in roles (parent to care giver), changes in health status (cognitive decline, disability, chronic conditions, etc.), which will cause some degree of derailment and changes in the purpose. A negative perception of ageing and fear of mortality also are contributory factors to the declining PIL [31]. Older adults, owing to their health status or other commitments will have varying challenges to fulfill the social roles that they were engaged in during their younger days that leads to a decline in PIL. Thus, the loss or changes in PIL in older adults is not only contributed by the age, but also is determined by other factors such as health, perceptions about ageing and life which acts in concert with each other. Likewise, marital status, education, ethnicity and gender are related to greater PIL. These factors play a role in securing employment, achieving social independence, active social and community level engagements which are all contributory factors to individual’s purpose. 

PIL is dynamic and changes during the course of life in response to various environmental and life circumstances. Individuals with a goal in mind would value them and pursue the goals despite the odds. In this journey, they will adopt behavioral changes to suit the role and accomplish efficient management of available resources. PIL is greater when there is a greater value attached to the tasks in pursuit. Greater PIL also balances the risk factors for various health conditions, thus having a life purpose improves life satisfaction and facilitates successful aging. Therefore, it is opined that PIL should be improved at every stage, especially when it seemed to decline. Community level activities targeting ageing communities, old age homes or similar settings could support their hobbies, engage them in social activities or other programs that improve health and well-being that will in turn foster a sense of purpose. Feeling them valued, getting them engaged in healthy lifestyle and social interactions should be considered in the process to improve the PIL of the older adults as this is the key to successful ageing. 

This systematic review has several strengths and limitations. To the best of our knowledge, this is the first review that looks at the conceptualization, measures and determinants of PIL in elderly and thus is a crucial piece of evidence to support the interventions for promoting PIL in elderly. This review also synthesized information from the qualitative study giving a better understanding of PIL. We have included grey literature and unpublished articles, such as theses, which could reduce the publication bias due to selective reporting in articles. Additionally, inclusion of both qualitative and quantitative evidence and exclusion of studies that do not report conclusive data (e.g., abstracts) improved the reliability of the evidence. Certain themes in the conceptualization, for example spirituality, were not explored in-depth due to the limited number of studies discussing those concepts. Together with the high heterogeneity in the conceptualization of PIL and measures employed, it discouraged a meta-analysis. We have omitted non-English articles therefore it is possible to omit relevant publications in other languages. The articles included in this review were mainly from the USA, Sweden, France, Canada, Australia, and Japan. Many other countries are not represented in the review. Not only this, but there were multiple articles by the same authors [53,54,55,57] from the same country. Thus, it is unclear if the conceptualization of PIL varies across geographical areas. Out of the 44 studies included in this systematic review, one study [47] had a RoB score of 25% (% of Yes for RoB checklist). The Spanish study did not report the study methodology clearly and how the confounders were handled that led to the rating. Nonetheless the outcome measures were valid and analysis was appropriate. Only twelve studies showed moderate RoB (five studies from the USA, three from Spain, and one each from Japan, Australia and Iran). The RoB did not appear to follow any observable pattern (e.g., study setting, design) and were mostly associated with confounding factors and how they were handles in the analysis. Thus, care should be taken while generalizing the findings from the study. Nevertheless, only a small subset of studies had moderate RoB and only a single study had high RoB which increase the confidence in the conclusion from this review. We have also excluded non-English articles from the review due to language barriers and limited resources which we could introduce a bias and reduce the generalizability of the results. Additionally, the systematic review mostly included cross sectional studies which allowed only unidirectional comparison of data. Thus, future studies should have a common understanding of PIL which will enable them to choose a more appropriate measure, allowing a meta-analysis of the evidence.

## 5. Conclusions

PIL is conceptualized in six different ways, two of which were the existing definitions of the construct. The majority of the measures had basic psychometric properties and relied on the existing definitions of PIL. This shows the complexity of the construct and the need for specific measures for older adults. Female gender, higher education, income, marital status and ethnicity, were associated with PIL. The included studies had low-moderate RoB and moderate strength of evidence in various domains. Thus, community-level interventions targeted to improve the PIL of older adults should be sensitive to the different ways the construct is understood by the population and factors that can influence the PIL. Future studies will benefit from the conceptual framework identified to understand the construct and select suitable instruments that are relevant for the population.

## Figures and Tables

**Table 1:** Study characteristics of the included studies and measures used.

No.	Study ID	Country	Setting	Design	Population	Number of Participants	Outcome Measures
1	Gerwood (1996) [39]	USA	Community, senior citizen centers	Cross sectional	Older adults 65 and above	n = 130	Demographic questionnaire, CEDS, and the 20-items Purpose in Life scale (by Crumbaugh and Maholick, PIL Test-C).
2	* Gerwood et al. (1998) [40]	USA	Community, senior citizen centers	Cross sectional	Older adults 65 and above	n = 130	Demographic questionnaire (for Spirituality) and PIL Test-C (20-items).
3	Bondevik and Skogstad (2000) [41]	Norway	Community and nursing home	Cross sectional	Older adults: 80 years and above	n = 110 for older adults from community; N = 111 those from nursing homes	Short Form Scale of the Revised UCLA Loneliness Scale; PIL test-C (20-items); A single item to measure religiosity (Would you say that religion means anything to you?)
4	Greenfield and Marks (2004) [42]	USA	Community	Cross sectional	Older adults 65–74 years	n = 373	Negative and positive affect scale; Ryff’s PWB Index (3-item); Major role-identity absences questionnaire.
5	Frazier et al. (2005) [43]	USA	Community: Senior centers, and public service organizations.	Cross sectional	Older adults 65 and above	n = 86	Multidimensional Measure of Religious Involvement for African Americans; Ryff’s PWB scale (14-item).
6	Nygren (2005) [44]	Sweden	Community	Cross sectional	Older adults 85 years of age or older	n = 125 n = 26 participants were 95 years or older, n = 46 were 90 years of age, and n = 53 were 85 years of age	The Swedish version of the PIL Scale-C (20-item); The Resilience Scale; SOC Scale: STS; SF-36 Health Survey.
7	Cicirelli (2006) [45]	USA	Community	Cross sectional	Older adults: young old group (60–74 years) and mid old group (75–84 years)	n = 192; young-old group n = 132 and mid-old group n = 60.	Self-rating of health, 2-items scale for Discrepancy between expected and desired time to live; PIL Scale-C (20-items); MFDS.
8	Dixon (2007) [46]	USA	Community (retirement community)	Cross sectional	Older adults above 70 years	n = 167	Interpersonal Mattering Scale; 20-item PIL Test-C; Short version of the Geriatric Depression Rating Scale; Older Adult Wellness Evaluation.
9	Triado et al. (2007) [47]	Spain	Community, retired individuals	Cross sectional study	Retired persons ages 65 and older	n = 422; n (men) = 200 n (women) = 222	Spanish version of the Ryff’s PWB Scale (9-items); Spanish version of Life Satisfaction Index; Philadelphia Geriatric Scale.
10	Moon and Mikami (2007) [48]	Japan	Community	Cross sectional	Older adults 65 years of age or older	n = 425 (n = 204 ethnic Korean residents; n = 221 for Japanese residents)	CGA for Activities of Daily Living, TMIG Index of Competence, short version of GDS-15 in Japanese and Korean visual horizontal analogue scale items to capture “sense of purpose in life”, self-reported medical history, and receipt of public assistance.
11	Ottenbacher et al. (2007) [49]	USA	Hospitalized inpatient sample (acute cases)	Cross sectional study	Older adults 65 years of age or older	n = 40	Ryff’s PWB scale (9-items)
12	Hedberg et al. (2009) [50]	Sweden	Community: town and rural areas	Qualitative	Women aged 85 and above	n = 30 women	PIL Test-C (20-items) for screening and the qualitative interview included various aspects of their lives, such as experiences of aging; difficult and positive life events; and experiences of loneliness, comfort, spirituality, and purpose in life.
13	Krause (2009) [51]	USA	Community	Cohort study	Older adults above 65 years	n = 1361	Informant report for mortality status; MIL scale (2-items for PIL); Self-rated health; Acute and chronic health conditions check list; Questions on Functional disability and Frequency of attendance at religious services; Emotional support scale.
14	Ferguson and Goodwin (2010) [52]	Australia	Community retirement villages, volunteer organizations and community organizations	Cross sectional	Older adults 65 to 94 years	n = 225	PIL subscale (14-items) of Ryff’s PWB scale; Affect Balance Scales (5-item subscale of Positive Affect scale); Revised Life Orientation Test (Dispositional optimism); The Social Support Questionnaire; Short Form. A scale (perceived control).
15	Hedberg et al. (2010a) [53]	Sweden	Community: town and rural areas	Longitudinal cohort	Elderly above 85 years	n = 149; women n = 88, men n = 61	GDS-15; OBS; OBS Scale; MADRS; DSM-IV; PIL Test-C (20-items).
16	Hedberg et al. (2010b) [54]	Sweden	Community: town and rural areas	Cross sectional	Elderly between 85 to 103 years of age.	n = 189; women n = 120, men n = 69	PIL Test-C (20-item); PGCM scale; GQL instrument; SF-36 Health survey; Katz Activity of Daily Living Index; sociodemographic questionnaire (social relations).
17	Hedberg et al. (2011) [55]	Sweden	Community: town and rural areas	Longitudinal study cohorts	Elderly above 85 years	n = 51 (42 women and 9 men)	PIL Test-C (20-item); GDS; The Minimal Nutritional Assessment; The Barthel Index for ADL (for PA), The Mini-Mental State Examination.
18	Lundman et al. (2012) [56]	Sweden	Community	Cross-sectional	Older adults 85 years and above	n = 185	The Swedish version of the PIL Scale-C; The Resilience Scale; SOC scale; STS scale; GDS-15; ADL scale; Self-reported measures for Living Arrangements and Social Relationships.
19	Hedberg et al. (2013) [57]	Sweden	Community: town and rural areas	Qualitative	Men above 85 years	n = 30	Interviews included questions about various aspects of their experiences of becoming and being very old.
20	Wilson et al. (2013) [58]	USA	Community (retirement communities, subsidized housing facilities, local churches, and social service agencies)	Cohort study	Older adults aged 65 years or older.	n (initial) = 1049 n (additional analysis) = 560	Ryff’s PWB scale (10-items; annually); Between 2008 and 2011, 18-item version of Ryff’s PWB Scales administered once to a subgroup; Cognitive tests for annual measures Episodic Semantic, working memory; Perceptual speed and Visuospatial ability; Guidelines of the joint working group of NINDS and SARDA; CESD; Katz disability scale.
21	Pearson et al. (2013) [59]	Australia	Community	Cohort study	Older adults aged 55 years of age or older	n = 545, 55–64 years n = 300, 65–74 years n = 167, 75 years and older n = 74	LET (6-items)
22	Zaslavsky et al. (2014) [26]	USA	Community	Cohort study	Women 85 years and older	n = 8880	All chronic conditions and disability (except diabetes) were diagnosed by a clinician while diabetes was captured based on self-report; Ryff PWB scale (7-item); Keyes scale for PG.
23	Windsor et al. (2015) [60]	Australia	Community	Cohort study Longitudinal	Older adults aged 65 and above.	n = 1475	PIL subscale from Ryff’s PWB scale (3-item); Items on Functional disability (sum of two mobility items and five items assessing difficulties with physical movement and lifting/ handling objects); Single item on self-rated health; CESD scale; Digit Symbol Substitution subscale of the revised WAIS (speed of processing); Boston Naming Task for episodic memory.
24	Tomioka et al. (2016) [61]	Japan	Community	Cohort study	Older adults aged 65 and older	n (total, mortality study) = 1853 n (ADL study) = 1556 n (IADL study) = 1399	Barthel index for ADL; TMIG Index of Competence; Single item for PIL; CPS for cognitive functions; GDS; Information about mortality, death and migration were obtained from the Shimoichi Town Hall; vital statuses of the participants were determined through the residential registration cards and death certificates.
25	Woods et al. (2016) [62]	USA	Community	Cohort study	Women 80 years and older	n = 26,704	ADL scale, major causes of morbidity, Perceived Health Scale. And questionnaire on Independent living and physical function for measuring successful aging; BRS; Self-Mastery; Confidence, Environmental Mastery and Self-control for effective aging; Optimal aging measured using emotional Well-being Scale and asking “Have you been happy” and “you enjoyed life most of the time”; Satisfaction with Life Scale, Positive Relations (Social Support Scale) and Satisfaction with Current QOL. Eudemonic well-being measured using PG subscale and PIL Subscale (8-items) from Ryff’s PWB Scales.
26	Sano (2016) [63]	Japan	Community, day-service centers	Cross-sectional	Older adults. No age limits specified. Mean age of participants was 77.1 + 8.7 years old	n = 281 n (male) = 127, n (female) = 154	SAMR and SOPI for Achievement motive; K-1 scale for PIL (16-items); Role expectation checklist.
27	Cook Maher et al. (2017) [64]	USA	Community	Longitudinal study cohorts	Older adults above 80 years	n = 50; Super agers n = 31, cognitively average older adults n = 19	Ryff’s PWB questionnaire (42-items).
28	Vanhove-Meriaux and Claude (2018) [65]	France	Community, non-nursing home	Cross-sectional study	Older adults, age 65 years and older	n = 182 n (women) = 102 n (men) = 80	Subscales of the French version of the Psychological Need Thwarting Scale—Older Adults; Psychological needs satisfaction was measured using three different questionnaires; French version of the Ryff’s PWB scales (14-items); French version of the subjective vitality scale; French version of the Rosenberg self-esteem Scale; French versions of the Positive and Negative Affect Schedule; French version of the GDS.
29	Musich et al. (2018) [66]	US	Community	Cross sectional	Older adults 65 years of age or older	n = 4563	NIH Tuberculosis Meaning and Purpose Scale Age 18+ (7-items) for PIL; BRS; ISEL; Self-reported measures on HL and reliance on faith; Health care utilization, compliance and expenditures measured through claims submitted, adherence to treatment protocol or medication adherence; Veteran’s RAND 12-item QOL scale.
30	Orang et al. (2018) [67]	Iran	Community	Cross sectional	Young (aged 17–25 years), middle-aged (aged 26–46 years), and older adults (aged 65–80 years)	n = 215 n = 84 young (aged 17–25 years), n = 59 middle-aged (aged 26–46 years), and n = 72 older adults (aged 65–80 years)	Stareg’s MIL Scale; Ryff’s PWB scale.
31	Polenick et al. (2018) [68]	USA	Community	Cross sectional	Older adults 65 years of age or older	n = 315	PIL subscale of Ryff’s PWB scale (1-item); Self-reports for Caregiving difficulties and gender.
32	Kim et al. (2019) [22]	South Korea	Community	Cross sectional	Older adults above 50; Age groups 50–59 years, 60–69 years, 70–79 years, 80 and above.	N = 11,525	PIL was measured through a modified scale created by combining five items from the Ryff Measures of Psychological Wellbeing and two additional items from Personal Growth and Self-Acceptance.
33	Oliveira et al. (2019) [69]	Brazil	Community	Cross sectional	Older adults 60 years of age or older	n = 92; n = 61 were 60–69 years old n = 31 were 70 years old or over	Sociodemographic questionnaire (use of medicine, patterns of PA, et c); The Satisfaction with Life Scale; Ryff PWB scale (10-item).
34	Poulos et al. (2019) [70]	Australia	Community	Mixed methods	Older adults 65 years of age or older	n = 127	Open-ended questions; WEMWBS; measures for Frailty; Focus group interviews
35	Sutin et al. (2020) [71]	USA	Community	Cross-sectional and longitudinal multi-cohort design	HRS: American and their spouses, 65 years and above NHATS: Participants aged 65 years and above from Medicare beneficiaries	Baseline sample: n = 6785 in HRS, n = 5665 in NHATS longitudinal sample: n = 4616 in HRS, n = 2877 in NHATS	HRS measured PIL using Ryff’s PWB scale (7-items) while NHATS used a single item; TICSm for cognitive function in HRS while sum of 3 tasks (memory, orientation and executive functions) in NHATS; HRS use 26 item version of MDI, NHATS use 10 item version of MDI; CESDS and PHQ-2; chronic conditions checklist of 7 conditions; Single items for PA.
36	Tkatch et al. (2020) [72]	USA	Community	Longitudinal cohort	Older adults above 65 years old	n (T1) = 216 n (T1 + 2) = 168 n (T1 + 2 + 3) = 125	HCC risk score for Clinical health status; Veteran’s RAND(VR-12); short version of the UCLE Loneliness Scale; BRS; NIH Tuberculosis Meaning and Purpose Scale Age 18+ (7-item); LOT-R for optimism.
37	Vanhove-Meriaux and Ferrand (2020) [73]	France	Community	Cross-sectional study	Older adults, above 65 years older	n = 154	French version of the PNFS-OA (BPNF); French version Ryff’s PWB scale (14 items); French version of the subjective vitality scale; PANAS; French version of the Rosenberg Self-Esteem.
38	Lewis et al. (2020) [74]	Canada	Community, Retirement individuals	Qualitative study	Older adults between the ages of 71 and 94 years (mean = 85.22 years)	n = 18	PIL sub-section of Ryff’s PWB scale (7-items); IADL Scale; Perceived Isolation Scale; GDS.
39	Lopez et al. (2020) [75]	Spain	Community	Cross sectional	Young-old (60–70 years) and Old-old (71–80 years).	n = 878	The Family APGAR; BRCS; Gratitude subscale of the Values in Action Inventory of Strengths-Short Form; AAQ-II; PG and PIL subscale of Ryff’s PWB scale (6-items).
40	Matud et al. (2020) [76]	Spain	Community	Cross sectional	Older adults 65 years of age or older	n = 1201	Spanish version of the Ryff’s PWB Scale (38-items (6 items for PIL)); scales of masculinity and femininity of the BSRI; Spanish version of the York Self-Esteem Inventory; Social Support Scale.
41	Bundick et al. (2021) [77]	USA	Community	Cross sectional	Older adults of 2 age groups 50–64 (midlife sample), and 65 and older (later life sample).	n = 1198; midlife sample: n = 799; later life sample n = 399.	WHO-HPQ; A new survey measure (10 items on a 5-point scale) for PIL and Purpose in Life (PIL) subscale of Ryff’s PWB scales (9-items); Empathic Concern subscale of Davis’s Interpersonal Reactivity Index (IRI). LGS for Generativity; GQ-6 for gratitude; Satisfaction with Life Scale; PGIS; BWSS.
42	Alfonso-Benlliure et al. (2021) [78]	Spain	Community	Cross sectional	Aged 65 years or older.	n = 152	MMSE for cognitive impairment; CESDS; TCI-A; Ryff’s PWB scale (items not reported).
43	Musich et al. (2021) [79]	USA	Community	Cross-sectional	At least 65 years of age with minimum of 12 month continuous medical plan enrolment (AARP Medicare Supplement Insured)	n = 3573	LET (6-items); BRS; LOT-R; Wallston’s MHLC scale; Social Network Index; PHQ-2; Veterans Rand-12 (VR-12); Healthcare utilization and expenditure captured from administrative medical claims as IP admissions or ER visits and paid medical claims within the one-year pre-survey period.
44	Zhang and Chen (2021) [80]	USA	Community	Cohort study	Older adults 65 years and older	n (T1) = 4591 n (T2) = 3687 n (T3) = 2818	Three items assessing the frequency of PA (vigorous, moderate and light-intensity PA); PIL subscale from Ryff’s PWB scale (7 items).

* Secondary analysis of Gerwood (1996). CEDS: The Centre for Epidemiologic studies Depression Scale; PIL Test-C: Purpose in Life scale (by Crumbaugh and Maholick); Ryff’s PWB scale: Ryff’s Psychological Well-Being scale; SOC scale: The Sense of Coherence Scale; STS: The Self-Transcendence Scale; MFDS: Multidimensional Fear of Death Scale; CGA: Comprehensive Geriatric Assessment; MIL: Meaning in Life; TMIG: Tokyo Metropolitan Institute of Gerontology Index; GDS-15: Geriatric Depression Scale; OBS scale: The Organic Brain Syndrome scale; MADRS: The Montgomery–Asberg Depression Rating Scale; DSM-IV: Diagnostic and Statistical Manual of Mental Disorders-IV; PGCM: The Philadelphia Geriatric Centre Morale; GQL: Gothenburg Quality of Life; PA: Physical Activities; ADL: Activities of Daily Living; PG: Personal Growth; WAIS: Wechsler Adult Intelligence Scale; NINDS: National Institute of Neurological Disorders and Stroke; SARDA: Stroke and the Alzheimer’s disease and Related Disorders Association; LET: Life Engagement Test; CPS: Cognitive Performance Scale; BRS: Brief Resilience Scale; SAMR: Scale for Achievement Motive in Rehabilitation; SOPI: Self-completed Occupational Index; NIH: National Institutes of Health; ISEL: Interpersonal Support Evaluation List, HL: Health Literacy; WEMWBS: Warwick–Edinburgh Mental Health and Well-Being Scale; HRS: Health and Retirement Survey; NHATS: National Health and Aging Trends Survey; TICSm: Telephone Interview for Cognitive Status, modified version; MDI: Midlife Development Inventory; PHQ: Patient Health Questionnaire; HCC: Hierarchal Condition Category; LOT-R: Life Orientation Test-Revised; BPNF: Basic Psychological Need Frustration; PNFS-OA: Psychological Need Frustration Scale for Older Adults; PANAS: Positive and Negative Affect Schedule; IADL: Instrumental Activities of Daily Living; BRCS: Brief Resilient Coping Scale; AAQ-II: The Acceptance and Action Questionnaire—II; BSRI: Bem Sex Role Inventory; WHO: World Health Organization; HPQ: Health and Performance Questionnaire; IRI: Interpersonal Reactivity Index; LGS: Loyola Generativity Scale; GQ-6: Gratitude Questionnaire-Six; PGIS: Personal Growth Initiative Scale; BWSS: Brief Wisdom Screening Scale; MMSE: Mini-Mental State Examination; TCI-A: Test of Creative Imagination for Adults; MHLC: Multidimensional Health Locus of Control.

**Table 2:** Citations and their conceptualization of PIL.

No.	Study ID	Health and Well-Being	Meaningful Aims and Goals	Inner Strength	Social Relationships	Mattering to Others	Spirituality and Religiousness
1	Gerwood et al. (1998) [40]						√
2	Bondevik and Skogstad (2000) [41]	√	√	√	√		√
3	Greenfield and Marks (2004) [42]		√		√		
4	Frazier et al. (2005) [43]		√	√	√		√
5	Nygren (2005) [44]	√		√		√	
6	Cicirelli (2006) [45]	√					
7	Dixon (2007) [46]	√		√	√	√	
8	Triado et al. (2007) [47]	√	√				
9	Moon and Mikami (2007) [48]	√					
10	Hedberg et al. (2009) [50]		√		√		√
11	Krause (2009) [51]	√	√		√		
12	Ferguson and Goodwin (2010) [52]	√		√			
13	Hedberg et al. (2010a) [53]	√	√				
14	Hedberg et al. (2010b) [54]	√	√		√		
15	Hedberg et al. (2011) [55]	√	√		√		
16	Lundman et al. (2012) [56]	√		√			
17	Hedberg et al. (2013) [57]	√	√	√	√	√	
18	Wilson et al. (2013) [58]	√	√				
19	Pearson et al. (2013) [59]		√				
20	Zaslavsky et al. (2014) [26]	√	√	√			
21	Windsor et al. (2015) [60]	√	√	√			
22	Tomioka et al. (2016) [61]	√					
23	Woods et al. (2016) [62]	√					
24	Sano (2016) [63]		√		√		
25	Cook Maher et al. (2017) [64]				√		
26	Vanhove-Meriaux and Claude (2018) [65]	√	√	√	√		
27	Musich et al. (2018) [66]	√		√	√		
28	Polenick et al. (2018) [68]	√	√				
29	Kim et al. (2019) [22]	√					
30	Oliveira et al. (2019) [69]	√			√		
31	Poulos et al. (2019) [70]	√	√	√	√		
32	Sutin et al. (2020) [71]	√	√				
33	Tkatch et al. (2020) [72]	√	√	√			
34	Vanhove-Meriaux and Ferrand (2020) [73]	√	√	√			
35	Lewis et al. (2020) [74]	√	√		√		
36	Lopez et al. (2020) [75]	√	√	√			
37	Matud et al. (2020) [76]	√	√	√	√		
38	Bundick et al. (2021) [77]		√			√	
39	Alfonso-Benlliure et al. (2021) [78]	√	√				
40	Musich et al. (2021) [79]	√	√	√	√	√	
41	Zhang and Chen (2021) [80]	√	√				

**Table 3:** Determinants of PIL.

No:	Study ID	Determinants
1	Gerwood (1996) [39]	Depression Spirituality
2	Gerwood (1998) [40]	Spirituality
3	Bondevik (2000) [41]	Religiousness Loneliness
4	Greenfield (2004) [42]	Role identity (volunteering moderate PIL and role identity)
5	Frazier (2005) [43]	Religious involvements
6	Nygren (2005) [44]	Resilience, sense of coherence, self-transcendence and perceived mental health
7	Cicirelli (2006) [45]	Fear of body loss Health
8	Dixon (2007) [46]	Overall wellness Mattering to others Depression
9	Triado (2007) [47] *	Personal growth Age Education Income
10	Moon (2007) [48]	Subjective well being
11	Ottenbacher (2007) [49]	Self-acceptance Positive Relation Environmental Mastery Personal Growth
13	Krause (2009) [51]	Self-rated Heath/fewer functional disabilities Mortality
14	Ferguson (2010) [52]	Optimism Perception of control (mediates relation between optimism and PIL)
16	Hedberg (2010) [53,54]	Attitude towards aging Having family Musculoskeletal symptoms
17	Hedberg (2011) [55]	Gender (women) Depression
18	Lundman (2012) [56]	Resilience, sense of coherence, self-transcendence
20	Wilson (2013) [58]	Global cognition/cognitive function
21	Pearson (2013) [59]	Education
22	Zaslavsky (2014) [26]	Health Disability Mortality
23	Windsor (2015) [60]	Memory Disability Decline of speed Health Depression Delayed mortality
24	Tomioka (2016) [61]	Higher mortality Instrumental activities of daily living
25	Woods (2016) [62]	Higher education Higher family income Marital status (married) Smoking status (non-smokers) Alcohol use (<1 drinks week)
26	Sano (2016) [63]	Achievement motive (Direct), Social participation Role expectation
28	Vanhove-Meriaux, (2018) [65]	High satisfaction need Low thwarting need Competence (extend to which people interact with environment) and relatedness need satisfactions (extend of a secure sense of belongingness and connectedness)
29	Musich (2018) [66]	High resilience High social support Strong alliance with faith High health literacy Good health Financial stress, living alone and >80 y had negative relation with PIL Reduced medical and drug expenditure Utilisation and expenditure Higher QoL Compliance with medication protocols Preventive service utilization
31	Polenick (2018) [68]	Gender specific fewer Emotional caregiving difficulties (females) Fewer physical care giving difficulties
32	Kim (2019) [22]	Higher cognition Decline in cognition
33	Oliveira (2019) [69]	Life satisfaction
35	Sutin (2020) [71]	Lower risk of concurrent motoric cognitive risk
37	Vanhove-Meriaux (2020) [73]	Basic psychological need frustration (predictor) Basic psychological need satisfaction
39	Lopez (2020) [75]	Perceived health, family functioning, resilience, gratitude and acceptance Loss of a loved one
41	Bundick (2021) [77]	Gender (female) Race/ethnicity Age
42	Alfonso-Benlliure (2021) [78]	Positive relationship with others Personal growth Environmental mastery Self-acceptance Life satisfaction Divergent thinking
43	Zhang and Chen (2021) [80]	Intensity of physical activity (predicted) Female gender, married, white/Caucasian, older at baseline, higher levels of education, better self-rated health, and fewer chronic conditions

* study has high risk of bias.

## Supplementary Materials

The following are available online at https://www.mdpi.com/article/10.3390/ijerph19105860/s1, Section S1: key words used, Table S1: Risk of Bias Assessments of Included Studies. Table S2: Questionnaires Used and Psychometric Properties.
