# Supplementary material for: Purpose in Life in Older Adults: A Systematic Review on Conceptualization, Measures, and Determinants
Source: Int J Environ Res Public Health. 2022 May 11;19(10):5860. doi: 10.3390/ijerph19105860 (PMC9141815; doi:10.3390/ijerph19105860)
Supplement: Supplementary file 1 [file ijerph-19-05860-s001.zip › ijerph-1689861-supplementary.pdf]

Supplementary Section S1

Keywords used

MEDLINE and Psych Info

1. "PIL".mp.
2. limit 1 to (english language and humans and yr="1995 -Current")
3. PIL.tw.
4. limit 3 to (english language and humans and yr="1995 -Current")
5. (purpose* adj3 life).mp.
6. limit 5 to (english language and humans and yr="1995 -Current")
7. PIL test.tw.
8. limit 7 to (english language and humans and yr="1995 -Current")
9. PIL questionnaire.tw.
10. limit 9 to (english language and humans and yr="1995 -Current")
11. exp "Surveys and Questionnaires"/
12. limit 11 to (english language and humans and yr="1995 -Current")
13. (Determinants, Epidemiologic or Epidemiologic Determinants or Health Social Determinants).mp.
14. limit 13 to (english language and humans and yr="1995 -Current")
15. determinants.mp.
16. limit 15 to (english language and humans and yr="1995 -Current")
17. ("65" and over).mp. [mp=title, abstract, original title, name of substance word, subject heading word, floating sub-heading word, keyword heading word, organism supplementary concept word, protocol supplementary concept word, rare disease supplementary concept word, unique identifier, synonyms]
18. limit 17 to (english language and humans and yr="1995 -Current")
19. (concept* adj3 purpose).mp.
20. limit 19 to (english language and humans and yr="1995 -Current")
21. (defin* adj3 purpose).mp.
22. limit 21 to (english language and humans and yr="1995 -Current")
23. ("older adults" or Geriatr*).mp. [mp=title, abstract, original title, name of substance word, subject heading word, floating sub-heading word, keyword heading word, organism supplementary concept word, protocol supplementary concept word, rare disease supplementary concept word, unique identifier, synonyms]
24. limit 23 to (english language and humans and yr="1995 -Current")
25. 65 year*.mp.
26. limit 25 to (english language and humans and yr="1995 -Current")
27. elderly.mp. or exp Aged/
28. limit 27 to (english language and humans and yr="1995 -Current")
29. (Age* adj3 "65").mp. [mp=title, abstract, original title, name of substance word, subject heading word, floating sub-heading word, keyword heading word, organism supplementary concept word, protocol supplementary concept word, rare disease supplementary concept word, unique identifier, synonyms]

30. limit 29 to (english language and humans and yr="1995 -Current")
31. 2 or 4 or 6
32. 18 or 24 or 26 or 28 or 30
33. 8 or 10 or 12
34. 14 or 16
35. 20 or 22
36. 33 or 34 or 35
37. 31 and 32 and 36
38. 31 and 32 and 36

Embase

1. ('PIL'/exp OR 'PIL') AND [humans]/lim AND [english]/lim AND [embase]/lim AND [1995-2021]/py
2. (concept* NEXT/3 purpose) AND [humans]/lim AND [english]/lim AND [embase]/lim AND [1995-2021]/py
3. (defin* NEXT/3 purpose) AND [humans]/lim AND [english]/lim AND [embase]/lim AND [1995-2021]/py
4. (purpose* NEXT/3 life) AND [humans]/lim AND [english]/lim AND [embase]/lim AND [1995-2021]/py
5. ('structured questionnaire'/exp OR 'survey'/exp OR 'qualitative research'/exp OR 'structured interview'/exp OR 'semi structured interview'/exp) AND [humans]/lim AND [english]/lim AND [embase]/lim AND [1995-2021]/py
6. ('determinants'/exp OR 'determinants') AND [humans]/lim AND [english]/lim AND [embase]/lim AND [1995-2021]/py
7. ('PIL questionnaire' OR 'PIL test') AND [humans]/lim AND [english]/lim AND [embase]/lim AND [1995-2021]/py
8. #1 OR #4
9. #2 OR #3
10. #5 OR #7
11. #6 OR #9 OR #10
12. #8 AND #11 AND 13

Web of science (Limits: LANGUAGE: (English) Indexes=SCI-EXPANDED, SSCI, A&HCI, CPCI-S, CPCI-SSH, ESCI Timespan=1995-2021)

1. (All="PIL”)
2. (AB= (concept* NEAR/3 purpose))
3. (AB= (purpose NEAR/3 life))
4. (AB= (defin* NEAR/3 purpose))
5. (ALL= (Questionnaire* OR Survey OR Rating scale OR Psycho* measure OR Psycho* assessment))
6. (ALL= PIL test)
7. (ALL=PIL questionnaire)
8. (AB= (determinants NEAR/3 purpose))
9. (ALL= (65 year$ OR elderly OR “older adults" OR geriatric$ OR "Frail elderly" OR "65 and older"))

10. (AB= (Age* NEAR/3 65)) #1 OR #3
11. #2 OR #4
12. #5 OR #6 OR #7
13. #9 Or #10
14. #12 OR #13 OR #8
15. #11 AND #15 AND #14

CINAHL (Limiters: Date: 19950101-20210131; English Language; Language: English Expanders – Apply equivalent subjects Search modes - Boolean/Phrase)

1. TX "PIL"
2. TX purpose* N3 life
3. TX PIL test
4. TX PIL questionnaire
5. (MH "Questionnaires+") OR (MH "Surveys+") OR (MM "Open-Ended Questionnaires") OR (MM "Structured Questionnaires") OR (MM "Survey Research")
6. (MM "Behavior Rating Scales") OR (MM "Psychological Tests") OR (MM "Body Weights and Measures") OR (MM "Attitude Measures") OR (MM "Outcome Assessment")
7. TX determinants
8. (MH "Older Adult Care (Saba CCC)") OR (MH "Geriatrics") OR (MH "Frail Elderly") OR "65 and older or geriatrics"
9. TX concept* N3 purpose
10. TX defin* N3 purpose
11. "65 year*"
12. (MH "Aged") OR "elderly or aged or older or elder or geriatric or elderly people or old people or old people or senior"
13. TX Age* N3 65
14. TX questionnaire or survey or scale or instrument or measurement or measure
15. (MM "Life Purpose") OR "PIL"
16. #1 OR #2 OR #15
17. #9 OR #10
18. #3 OR #4 OR #5 OR #6 OR #14
19. #8 OR #11 OR #12 OR #13
20. #7 OR #17 OR #18
21. #16 AND#19 AND #20

Table S1. Risk of Bias Assessments of Included Studies.

Citation	Study Design	Q1	Q2	Q3	Q4	Q5	Q6	Q7	Q8	Q9	Q10	Q11	Overall	% of Yes	ROB
Gerwood (1996)	Cross sectional	Y	Y	Y	Y	N	Y	Y	Y	-	-	-	Include	87.5	Low
Gerwood (1998)	Cross sectional	Y	Y	Y	Y	N	Y	Y	Y	-	-	-	Include	87.5	Low
Bondevik (2000)	Cross sectional	Y	Y	Y	Y	N	N	Y	Y	-	-	-	Include	75	Low
Greenfield (2004).	Cross sectional	Y	Y	Y	Y	N	N	Y	Y	-	-	-	Include	75	Low
Frazier (2005)	Cross sectional	Y	Y	Y	Y	N	N	Y	Y	-	-	-	Include	75	Low
Nygren (2005)	Cross-sectional	Y	Y	Y	Y	N	N	Y	Y	-	-	-	include	75	low
Cicirelli (2006)	Cross sectional	N	Y	Y	Y	N	N	Y	Y	-	-	-	Include	62.5	Moderate
Dixon (2007)	Cross sectional	N	Y	Y	Y	N	N	Y	Y	-	-	-	Include	62.5	Moderate
Triado (2007)	Cross-sectional	N	N	Y	NA	N	NA	Y	Y	-	-	-	Include	25	High
Moon (2007)	Cross-sectional	Y	Y	Y	Y	N	N	Y	Y	-	-	-	include	75	low
Ottenbacher (2007)	Cross-sectional	UC	Y	Y	Y	N	N	Y	Y	-	-	-	include	62.5	moderate
Hedberg (2009)	Qualitative	Y	Y	Y	Y	Y	N	N	Y	Y	Y		include	80	Low
Krause (2009).	Cohort study	Y	Y	Y	Y	Y	Y	Y	Y	N	N	Y	include	81.8	Low
Ferguson (2010)	Cross sectional	N	Y	Y	Y	Y	Y	Y	Y	-	-	-	Include	87.5	Low
Hedberg (2010)	Longitudinal cohort	Y	Y	Y	Y	Y	Y	Y	Y	Y	N	Y	Include	90.9	Low
Hedberg (2010)	Cross sectional	Y	Y	Y	Y	N	Y	Y	Y	-	-	-	Include	87.5	Low
Hedberg (2011)	Longitudinal cohort	Y	Y	Y	N	N	NA	Y	Y	Y	N	Y	Include	70	Moderate
Lundman (2012)	Cross-sectional	Y	Y	Y	Y	N	N	Y	Y	-	-	-	include	75	low
Hedberg (2013)	Qualitiative study	Y	Y	Y	Y	Y	N	N	Y	Y	Y		include	80	Low
Wilson (2013)	Cohort study	Y	Y	Y	Y	Y	Y	Y	Y	Y	Y	Y	Include	100	low
Pearson (2013)	Cross-sectional	Y	UC	Y	Y	N	N	Y	Y	-	-	-	include	62.5	moderate

Zaslavsky (2014)	Cohort study	Y	Y	Y	Y	Y	N	Y	Y	Y	Y	Y	Include	90.9	Low
Windsor (2015)	Cohort study	Y	Y	Y	Y	Y	N	Y	Y	Y	Y	Y	Include	90.9	Low
Tomioka (2016)	Cohort study	Y	Y	N	Y	Y	N	Y	Y	Y	Y	Y	Include	81.8	Low
Woods (2016)	Cohort study	Y	Y	Y	N	NA	Y	Y	Y	UC	N	Y	Include	63.6	Moderate
Sano (2016)	Cross-sectional	UC	N	Y	Y	N	N	Y	Y	-	-	-	include	50	moderate
Cook Maher (2017)	Longitudinal cohort	Y	Y	Y	Y	Y	Y	Y	NR	Y	NR	Y	Include	83.3	Low
Vanhove-Meriaux (2018)	Cross-sectional	Y	N	Y	N	Y	Y	Y	Y	-	-	-	Include	75	Low
Musich (2018)	Cross-sectional	Y	Y	Y	Y	Y	Y	Y	Y	-	-	-	include	100	low
Orang (2018)	Cross-sectional	UC	UC	Y	Y	N	N	Y	Y	-	-	-	include	50	moderate
Polenick (2018)	Cross-sectional	Y	Y	Y	Y	N	N	Y	Y	-	-	-	include	75	low
Kim (2019)	Cross sectional	N	N	Y	Y	Y	Y	Y	Y	-	-	-	include	75	Low
Oliveira (2019)	Cross-sectional	Y	Y	Y	Y	N	N	Y	Y	-	-	-	include	75	low
Vanhove-Meriaux (2020)	Cross-sectional	Y	N	Y	Y	N	Y	Y	Y	-	-	-	Include	75	Low
Lewis (2020)	Qualitative	Y	Y	Y	Y	Y	Y	N	N	Y	Y	-	include	80	low
Lopez (2020)	Cross-sectional	Y	N	Y	Y	N	N	Y	Y	-	-	-	include	62.5	moderate
Matud (2020)	Cross-sectional	Y	N	Y	Y	N	N	Y	Y	-	-	-	include	62.5	moderate
Bundick (2021)	Cross sectional	N	Y	UC	Y	Y	Y	Y	N	-	-	-	Include	62.5	Moderate
Sutin (2021)	Cross-sectional and longitudinal	N	Y	Y	Y	Y	Y	Y	Y	-	-	-	Include	87.5	Low
Tkatch (2021)	Longitudinal/ observational cohort	Y	Y	Y	Y	Y	N	Y	Y	Y	N	Y	Include	81.8	Low
Alfonso-Benlliure (2021)	Cross-sectional	Y	N	Y	Y	N	NA	Y	Y	-	-	-	Include	62.5	Moderate
Musich (2021)	Cross-sectional	Y	Y	Y	Y	Y	Y	Y	Y	-	-	-	Include	100	low

Zhang (2021)	Cohort study	Y	Y	Y	Y	Y	N		Y		Y		Y		Y		Y	Include	90.9	low
Poulos (2019) *	Mixed methods	Y	Y	Y	Y	Y	Y	Y	Y	Y	Y	UC	Y	Y	Y	UC	Y	Include	82.3	Low

* Using Mixed methods appraisal tool for Risk of bias assessments.

Table S2. Questionnaires Used and Psychometric Properties.

Study ID (author name and year)	Questionnaire Used	Main Results	Items	Psychometric Properties
Greenfield and Marks (2004)	Ryff's PIL Index	The adapted scale correlated highly (r > 0.70) with the parent 20-item scale.	3-items	The adapted scale correlated highly (r >.70) with its 20-item parent scale.
Frazier et al (2005)	Ryff's PWB scale	PIL scores were 71.6 (SD 9.72)	14-item	The correlation of the shortened version of the Ryff's well-being scale to that of the parent scale ranged from 0.97 to 0.98.
Triado et al (2007)	Ryff's PWB scale (Spanish version)	The internal consistency coefficients of the subscales were modest to low. Exploratory principal component analysis and confirmatory factor analysis did not support the 6-factor structure indicating a reduction in internal consistency and validity of the scale.	9-items	Cronbach α for Autonomy sub scale was 0.63<br>Cronbach α for Positive relations subscale was 0.65.<br>Cronbach α for Self-acceptance subscale was 0.65.<br>Cronbach α for Environmental mastery PG and PIL subscales were 0.71, 0.66, and 0.58, respectively.
Ottenbacher et al (2007)	Ryff's PWB instrument	PIL score<br>(a) Mean score at admission was 39.3 (±8.3)<br>(b) Mean score at discharge was 37.4 (±9.3) p = 0.34	9-items	ICC of test-retest reliability ranged from 0.66-0.79 across the six domains of PWB. ICC of PIL domain was 0.79, p < 0.01. Good internal consistency for the 6 domains was demonstrated (Cronbach's α's from 0.86-0.93). Test-retest reliability with Pearson

				product moment coefficients over a six-week period ranged from 0.81-0.88.
Ferguson and Goodwin (2010)	PIL sub-scale from Ryff's PWB instrument	PIL score in the study sample was mean: 66.6 (±10.5)	14 items	Cronbach's coefficient was 0.88.
Wilson et al (2013)	Ryff's PWB scale	(a). Global cognition and PIL were negatively related to age ($r = -0.28$, $p < .001$ for cognition; $r = -0.24$, $p < 0.001$ for purpose) and positively related to both education ($r = 0.27$, $p < .001$ for cognition; $r = 0.38$, $p < 0.001$ for purpose) and one another ($r = 0.29$, $p < 0.001$).<br>(b) Men reported a slightly higher PIL (M = 3.70, SD = 0.47) than did women (M = 3.59, .SD = 0.46; $p < 0.001$.) implying that gender has a role in determining purpose.<br>(c) Cognitive decline was more strongly related to purpose than to self-acceptance and autonomy (Less cognitive decline was associated with higher PIL).	10-items (Phase 1, 2001)<br>18-items (Phase 2, 2008-2011)	Cronbach's coefficient (10-items) was 0.74, indicating an adequate level of internal consistency.
Zaslavsky et al (2014).	Ryff's PWB scale	Women in Healthy group had higher PG and PIL scores than those in other groups. Both the constructs were independently associated with late life risk of death and higher odds of having a mobility disability and major chronic morbidity before the age of 85 years. women who reported lower PIL or PG score had higher risk of death in the 2-year study period.	7 items	The modified PIL measures used for this analysis had acceptable reliability (Cronbach's α's of 0.65)
Windsor et al (2015).	PIL Scale from Ryff's Scales of PWB: "I have a sense of direction and PIL"; "I used to set goals for myself, but now that seems like a waste of time"	(a) The three items from Ryff's PIL Scale were validated in this study. They loaded significantly and positively on a single factor (standardized loadings ranged from 0.44 to 0.72). Further multiple group CFA also determined that this measure exhibited measurement invariance across young-old (65-76) and older adults (77-105) ($x2(4) = 1.96$, $p = 0.74$; CFI =1.00; RMSEA = 0.00).	3 items	Cronbach's α (a = 0.58) was modest but compared favorably with the three-item purpose measure from Ryff's original scale.

	(reversed); and "I enjoy making plans for the future and working to make them a reality."	(b) Correlations of 0.36 (perceived control) and 0.34 (self-esteem) indicated that the purpose measure is related to both control beliefs and self-esteem, but does not directly assess either construct, supporting convergent validity. (c) Individuals with higher sense of purpose had lower levels of disability $p < 0.05$), better performance on tests of speed $p < 0.05$) and memory $p < 0.05$),better self-rated health $p < 0.05$) and fewer depressive symptoms $p < 0.05$). (d) High scores in PIL was associated with marginally slower rates of decline of speed $p < 0.05$) over the study interval. (e) High PIL was associated with better probability of survival, however the association weakened over time.		
Woods et al (2016).	PIL Subscale from Ryff's PWB scale	(a) Average score on PIL Scale was 18 and average scores ranged from 17 to 19 across various demographic groups. (b) High levels of optimal aging were reported in the study sample.	8 items	NR
Cook Maher et al (2017)	Ryff's PWB scale	(a) Super Agers endorsed a significantly greater level of Positive Relations and episodic memory than cognitively average adults. (b) No significant differences in PIL between the super agers and cognitively average older adults.	42 items	NR
Vanhove-Meriaux and Claude (2018).	French version of the Ryff's PWB scale	(a) Those with high psychological satisfaction-low thwarting had a mean PIL score of 3.75 (±0.4) while those with moderate psychological satisfaction- moderate thwarting) had mean of 3.42 (±0.46). (b) For PIL, only competence BPNS was a significant predictor ($p = 0.048$), the other BPNS and BPNT measures were not significant.	14 items	NR

Orang et al (2018)	Ryff's PWB scale	(a) PIL score<br>Age 17-25years group mean: 9.90 (±2.52)<br>Age 26-46years group mean:10.02 (±2.87)<br>Age 65-80years group mean:12.27(±3.09)<br>(b) There was a significant increase in PIL with age. The mean score of purposeful life for the age group of 65-80 years was statistically significantly more than the other two age groups.	NR	The coefficient of test-retest reliability was 82%. The reliability coefficients of the subscales of acceptance, the positive relationship with others, self-determination, objective life, personal growth, and environmental domination were 71%, 77%, 78%, 70%, 77%, and 78%, respectively.
Polenick et al (2018)	Ryff's PWB scale	(a) Caregivers' experiences of PIL were significantly negatively correlated with emotional ($r = -0.27$, $p < 0.001$) and physical ($r = -0.24$, $p < 0.001$) caregiving difficulties.<br>(b) There were no significant differences between caregiving wives and husbands in caregiver or care recipient reports of PIL.	1-item	NR
Oliveira et al (2019)	Ryff PWB scale	(a) The elderly had a good degree of both, life satisfaction (Median (Md) = 29.0) and PIL.<br>(b) A statistically significant greater PIL score (Md = 34.5) was seen in elderly who had been practicing hydro gymnastics for a duration of 1 to 5 years.<br>(c) Elderly with good self-perceived health had significantly higher scores on the PIL scale ($p = 0.047$).	10-items	NR
Vanhove-Meriaux and Ferrand (2020).	Ryff's PWB scales	(a) PIL scale had a Cronbach α of 0.71 and a Mean of 3.58 (±0.49).<br>(b) Participants reported moderate levels of personal growth (Mean: 3.52 (±0.54) and PIL (Mean: 3.58 (± 0.49).	14 items	Cronbach α was 0.71
Lewis et al (2020)	Sub-section of Ryff's PWB Scale and semi structured interviews	(a) Mean purpose levels were comparable to other samples of community-dwelling and oldest old adults.<br>(b) PIL mean score was 4.25 (±0.83).	7-items	NR

		(c) Community activities, social opportunities within the facility, availability of nursing care and other supports, contributed to the PIL of the older adults.<br>(d) Declining health, imminent death, adjustments to the new environment, and lack of control over decisions to move, was perceived as a barrier to participants' PIL.		
Lopez et al (2020)	Spanish Version of Ryff's PWB scale	(a) Young-old participants didn't experience more PIL ($p = 0.726$) than old-old participants, but experienced more personal growth.<br>(b)Young-old PIL mean:28.00 (±4.62) Old-old PIL mean: 28.12 (±4.93).<br><br>(c) The old-old group reported more gratitude and resilience ($p < 0.05$).	6-items	Reliability for PIL, Cronbach's $\alpha = 0.808$.
Matud et al (2020)	Spanish version of the Ryff's PWB scale	(a) Mean PIL score in men was 27.62 (±5.14), and for women was 26.43 (±5.61).<br>(b) Men reported higher scores than women in PIL, self-acceptance, autonomy, environmental mastery, and in the overall well-being score, though the effect sizes were noted to be small.	38-items (6 items for PIL)	Internal consistency is 0.82 for current sample.
Alfonso-Benlliure et al (2021)	Ryff's PWB scale	A significant relationship was noted between divergent thinking and PWB and the latter with life satisfaction. There was no direct relationship between divergent thinking and life satisfaction.	NR	PIL ($\alpha = 0.75$).
Zhang and Chen (2021)	Ryff PWB scale	(a) A high level of PIL was associated with more engagement in different intensities of PA in the future (PIL Mean: 31.05 (±6.60) at T1, 31.66 (±6.07) at T2, 30.64 (±6.70) at T3). The PA variables did not predict subsequent PIL.	7-items	Cronbach's α for Ryff Scales of Psychological Well Being was 0.72 at T1, 0.76 at T2 and 0.75 at T3.

Kim et al (2019)	Modified new scale created by combining five items from the Ryff Measures of PWB and two additional items from Personal Growth and Self-Acceptance.	(a) Those who were 70-70 years and 80 or above had lower PIL than younger age group.<br>(b) Participants who were black and of other ethnicities and who earned at least a college degree showed greater PIL than did their counterparts.<br>(c) PIL was positively associated with cognitive scores and moderated the relationship between age, ethnicity and cognitive decline.<br>(d) PIL was protective for cognitive decline in those who are old and from black ethnicity but had no interaction with gender.	7 items	The Cronbach's α for the 7-item PIL scale was 0.76.
Sutin et al (2020)	**Data from 2 studies** NHRS measured PIL with 7-item PIL scale from the Ryff Measures of PWB scale. NHATS measure PIL using a single item "My life has meaning and purpose".	(a) PIL was significantly associated with 33% and 26% lower risk of concurrent and incident Motoric cognitive risk.<br>(b) The baseline PIL scores were 4.6 (NHS) and 2.83 (NHATS)	7 items<br>1-item	Cronbach's α of the NHS questionnaire was= 0.74.
Bundick et al (2021)	A new survey measure (10 items on a 5 point scale) and PIL (PIL) subscale of Ryff's PWB scales (9-items, 7 point scale	(a) 30.97% of the overall study participants met the criteria for purpose, and 69.03% did not have a purpose.<br>(b) 29.79% of the midlife subsample and 33.33% of the later life sample met the criteria for purpose. (.	9 items<br>10 items	The reliability for the subscale in the current study was 0.84.
Gerwood (1996)	PIL Test-C	(a) The overall mean PIL score was 109.85 (±17.93; males: 110.56 (±=18.95); Females: 109.21 (±17.06).<br>(b) Higher depression was negatively associated with PIL.	20 items	The Spearman-Brown corrected rating for modified PIL test was 0.92; Two questions were dropped in the revised instrument from the original test.

Gerwood et al (1998), secondary data	PIL Test-C	(a) No significant differences were noted in the PIL raw scores between Protestants (Mean:110.15 (±18.71) and Catholics (Mean:110.93 (±16.44).<br>(b) No differences between Protestants and Catholics in the meaningfulness of spirituality ($p > 0.05$).	20-items	The published values for test–retest reliability coefficient was .83 ($p< .01$) in a sample of church parishioners
Bondevik and Skogstad (2000)	PIL Test-C	(a) Nursing home residents had significantly lower PIL than community dwelling elderly (93.9 (±16.9 vs 99.9 (SD = ± 20; $p < 0.01$). No age group differences were noted.	20 items	The Cronbach α coefficient for the PIL scale was 0.90.
Nygren (2005)	The Swedish version of the PIL Test-C	There was a statistically significant difference in mean PIL scores between women and men, where men had higher scores (110 (±13.3) versus 103 (±16.3), $p = 0.017$). However, there were no significant differences between the age groups in terms of mean scores.	20-items	Concept and concurrent validity are reported to have been well established, and it has a test-retest reliability of 0.83. In this study, the reported Cronbach's α was 0.85.
Cicirelli (2006)	PIL Test-C	(a) PIL (46.7 vs 49.4) and self-reported health (4.2 vs 4.7) was significantly less in mid-old group than young-old group ($p < 0.05$).<br>(b) PIL and difference between expected and desired time remaining to live had indirect effect on health but direct effect on fear of body loss.	9-items	The study employed a modified version of Crumbaugh's PIL scale where the original scale (20 items, 7-point scale; internal consistency 0.85) was modified to a subset of 9 items (internal consistency of 0.79).
Dixon, (2007)	PIL Test-C	Mean score for PIL was 5.1 (±0.93)	20-items	The Cronbach's α was 0.94 for the total sample.
Hedberg et al (2010)a	PIL Test-C	.<br>(b) At baseline, those with depression had lower PIL score (mean: 107 (±15) vs. mean: 99 (±15), $p=0.014$).<br>(c) Women had significantly lower PIL scores compared to men (mean: 103 (±16) vs. mean: 109 (±13) $p = 0.02$).<br>(d) The mean PIL score at baseline did not differ between the	20 items	The Cronbach's α was 0.84

		groups who were diagnosed five years later as either not depressed (mean: 108 (±16)) or depressed (mean: 106, (±17), p = 0.750.		
Hedberg et al (2010)b, secondary data	PIL Test-C	(a) The mean PIL scores for the total sample was 105 (±15.8); range 58 to 140). Women scored lower on this PIL scale than men (102 vs.108 p = 0.013).<br>(b) Attitudes towards aging, having someone to talk to and musculoskeletal symptoms were associated with PIL.	20 items	The Cronbach's α was 0.84.
Hedberg et al (2011), secondary data	PIL Test-C	(a) There was a significant reduction in PIL scores after 5 years compared to baseline (108, (±16); 103, (±15); p = 0.002).<br>(b) The reduction in PIL over 5 years were significant in females (PIL I mean: 106 (±16); PIL II m = 100, SD = 15; p = 0.007) but not in males (PIL I mean: 115 (±12); PIL II mean: 113, (± 8); p = 0.82).<br>(c) There were no significant differences in PIL scores at baseline for those who living alone vs living with someone or those with a diagnosis of depression vs without depression, however, at follow up those with depression and those living alone showed a significant drop in PIL scores (p = 0.001).<br>(d) There was no association with PIL and risk for developing depression at follow up.	20 items	Cronbach's α for PIL test in the current study was 0.84.
Lundman (2012)	The Swedish version of the PIL Test-C	(a) Mean PIL score was 105.0 (±15.9).<br>(b) RS, SOC, STS, and PIL scales showed fair to good inter-correlation and good correlation with the inner strength sum score.	20 items	Cronbach's α was 0.85.
Pearson et al (2013)	LET	(a) At both time points T1 and T2 (12 months apart), most participants reported a high level of PIL, with LET scores reflecting a small increase from T1 to T2.	6 items	The scale demonstrated sound internal consistency (Cronbach's a 0.84 and 0.82 at

		(b) LET mean (SD)<br>(i) 55–64years T1 24.89 (±4.21), T2 25.45 (±3.81)<br>(ii) 65–74years T1 25.10 (±4.20), T2 25.74 (±3.78)<br>(iii) 75years and older T1 24.30 (±3.85), T2 24.44 (±3.68)<br>Higher scores indicate higher PIL		T1 and T2, respectively).Results for longitudinal invariance were inconclusive.
Musich et al (2021)	LET	(a) The population prevalence of the five high protective factors were: purpose-in-life (64%), resilience (43%), optimism (26%), internal LOC (28%) and social connections (26%).<br>(b) The number of high Purpose-in-Life score is significantly correlated with the number of Protective Factors ($p < 0.0001$)<br>(c) The number of protective factor positively predicted health outcomes.<br>(d) Significant reductions in healthcare utilization and expenditures ($p = 0.002$) were noted. Utilization of ER visits ($p = 0.04$) and IP admissions ($p = 0.07$) were reduced as the number of protective factors increased.	6-items	NR
Musich et al (2018)	NIH Tuberculosis Meaning and Purpose Scale Age 18+	Among the participants, the prevalence of low, medium, and high PIL levels was 24.2%, 21.1% and 54.7% respectively	7-items	NR
Tkatch et al (2020)	NIH Tuberculosis Meaning and Purpose Scale Age 18+.	(a) There was significant improvement in PIL from T1 (M 26.08) to T2 (M27.17, $p = 0.01$). This relationship remained significant ($p = 0.01$) after controlling for gender. Loneliness decreased for the same period.<br>(b) The greater time spent interacting with the pets was associated with an improvement in participants' mental health ($p = 0.05$) with a marginal effect on optimism ($p < 0.10$) at T3.	7-items	This questionnaire was validated within the NIH study with a Cronbach α of 0.84. In this study, Cronbach's α for purpose tested was 0.93).

Sano (2016)	K-1 scale	(a) In a test of normality of each scales, total score of K-1 Scale was 0.000 (skewness = -0.55, kurtosis = -0.51). (b) All items for the SAMR, SOPI, and K-1 Scales were accepted, satisfying the standard of PCC. (c) CFA of SAMR, SOPI, and K-1 Scale demonstrated good fit with statistics of the same structure with previous studies. (d) Achievement motive had a direct effect on PIL ($p < 0.001$), indirect effect on PIL via social participation or role expectation ($p < 0.001$) and a total effect on PIL (total effect D 0.62).	16-items	See main results.
Moon and Mikami (2007)	Questionnaire to measure absence of sense of PIL	(a) The ethnic Korean elderly were more likely to suffer a lack of sense of PIL compared to their Japanese peers and (27% vs 16.1%) had a higher prevalence of diabetes, heart conditions and hypertension. (b) Ethnicity was a significant variable for well-being.	NR	NR
Krause (2009)	PIL was measured using an abbreviated version of the MIL scale created by the authors from other published scales. The scale measured and it measured each of four dimensions of MIL: having values, a sense of purpose, goals, and the ability to reconcile things that have happened in the past, and chose two	(a) Older adults with a greater sense MIL were less likely to die than older people who do not have a greater sense of MIL (OR = 0.92; $p < .001$). (b) Older adults who rated their health better were less likely to die than older people who report that their health is not as good (OR = 0.72; $p < 0.01$). (c) Older adults with greater functional disability had an increased mortality risk (OR = 1.11; $p < 0.001$). (d) The effect of MIL on mortality operated through health (e) PIL had the strongest relationship with mortality than other facets of MIL.	2-items	The correlation between this abbreviated version and the long version of the MIL scale was 0.98($p < .001$). The internal consistency estimate was 0.86 at Wave 4, and 0.9 at Wave 5.

	indicators that best measured each of these dimensions from a CFA (data not reported).			
Tomioka et al (2016)	PIL was measured by yes or no to "Do you have Ikigai". (not validated)	(a) 62.4% of the sample had hobbies and PIL, 16.4% had PIL only, 7.4% had hobbies only and 13.9% had none.<br>(b) Absence of PIL and hobbies was significantly associated with mortality (hazard ratio: 2.08, 95% CI: 1.47–2.94) ADL (2.74; 95% CI, 1.44–5.21) and IADL (OR 1.89; 95% CI, 1.01–3.55)	1 item	NR

PIL: Purpose in Life; PWB: Personal Well-Being; MIL: Meaning in Life; PG: Personal Growth; ICC: intraclass correlation; PA: Physical Activity; PIL Test-C: PIL Test by Crumbaugh & Maholick; LET: Life Engagement Test; NIH: National Institutes of Health HRS: Health and Retirement Survey; NHATS: National Health and Aging Trends Survey; RS: Resilience Scale; SOC: Sense of Coherence; STS: Self Transcendence; LOC: locus of control; SOPI: Self-completed occupational index; PCC: Polyserial Correlation Coefficient ; ADL: Activities Of Daily Living; IADL: Instrumental Activities of Daily Living; CFA: Confirmatory Factor Analysis
